# A gait abnormality measure based on root mean square of trunk acceleration

**DOI:** 10.1186/1743-0003-10-118

**Published:** 2013-12-26

**Authors:** Masaki Sekine, Toshiyo Tamura, Masaki Yoshida, Yuki Suda, Yuichi Kimura, Hiroaki Miyoshi, Yoshifumi Kijima, Yuji Higashi, Toshiro Fujimoto

**Affiliations:** 1 Faculty of Biomedical Engineering, Osaka Electro-Communication University, 18-8 Hatsucho, Neyagawa, Osaka 572-8530, Japan; 2 Graduate School of Engineering, Chiba University, Chiba, Chiba, Japan; 3 Faculty of Biology Oriented Science & Technology, Kinki University, Kinokawa, Wakayama, Japan; 4 Sharp Corporation, Tenri, Nara, Japan; 5 Fujimoto General Hospital, Miyakonojyo, Miyazaki, Japan

**Keywords:** Gait abnormality measure, Trunk acceleration, Root mean square (RMS) ratio

## Abstract

**Background:**

Root mean square (RMS) of trunk acceleration is seen frequently in gait analysis research. However, many studies have reported that the RMS value was related to walking speed. Therefore, the relationship between the RMS value and walking speed should be considered when the RMS value is used to assess gait abnormality. We hypothesized that the RMS values in three sensing axes exhibit common proportions for healthy people if they walk at their own preferred speed and that the RMS proportions in abnormal gait deviate from the common proportions. In this study, we proposed the RMS ratio (RMSR) as a gait abnormality measure and verified its ability to discriminate abnormal gait.

**Methods:**

Forty-seven healthy male subjects (24–49 years) were recruited to examine the relationship between walking speed and the RMSR. To verify its ability to discriminate abnormal gait, twenty age-matched male hemiplegic patients (30–48 years) participated as typical subjects with gait abnormality. A tri-axial accelerometer was attached to their lower back, and they walked along a corridor at their own preferred speed. We defined the RMSR as the ratio between RMS in each direction and the RMS vector magnitude.

**Results:**

In the healthy subjects, the RMS in all directions related to preferred walking speed. In contrast, RMSR in the mediolateral (ML) direction did not correlate with preferred walking speed (*rs = -0.10*, *p = 0.54*) and represented the similar value among the healthy subjects. Moreover, the RMSR in the ML direction for the hemiplegic patients was significantly higher than that for the healthy subjects (*p < 0.01*).

**Conclusions:**

These results suggest that the RMSR in the ML direction exhibits a common value when healthy subjects walk at their own preferred speed, even if their preferred walking speed were different. For subjects with gait abnormality, the RMSR in the ML direction deviates from the common value of healthy subjects. The RMSR in the ML direction may potentially be a quantitative measure of gait abnormality.

## Background

Recently, accelerometry has been widely used to study human body movement as an alternative approach to conventional motion analysis techniques, such as optoelectronic and force plate motion analyses. The benefits of accelerometers are as follows: low cost compared with commonly used motion analysis equipment; measurement not being limited to a laboratory environment; and accelerometers being small in size and lightweight, which avoids interference with a subject’s movement [1]. Typical applications of accelerometry are gait analysis, balance evaluation and screening of falling risk [2-17]. By using the characteristics of acceleration patterns, several researchers have proposed algorithms to obtain spatiotemporal parameters such as walking cycle, step duration, stance and swing durations [2,3]. Gait abnormality and falling risk have also been assessed by applying frequency analysis including harmonic analysis [2,4-8], time-frequency analysis [9,10], non-linear analysis such as entropy analysis [11,12] and fractal analysis [13,14] to body acceleration.

Root mean square (RMS) of acceleration is also seen frequently in gait analysis research [3-8,11,12,15-17]. This parameter constitutes a statistical measure of the magnitude of acceleration. Computation of the RMS is extremely simple and requires no preconditions like an optimal threshold and accurate peak detection to obtain characteristics of the signal pattern. Thus, the physical meaning of an RMS value is clear, and the value is easy to use in clinical practice. Moe-Nilssen showed that the RMS value along the mediolateral direction in a slightly balance-impaired subject increased compared with that in normal subjects [4]. On the other hand, Mizuike et al. demonstrated that RMS values of all three directions in stroke patients were significantly lower than those of normal subjects [16]. It is expected that one of the main factors of the discrepancy in these findings for abnormal gait comes from the relationship between RMS and walking speed. Moe-Nilssen studied the difference of RMS between normal and abnormal walking at the same walking speed [4]. Mizuike et al. evaluated the RMS of healthy subjects and stroke patients at their preferred walking speed [16]. In these studies, the conditions of walking speed differed. Many studies reported that the RMS had a high correlation with walking speed [3-6,11,15]. Therefore, Menz et al. and Latt et al. suggested that the usefulness of RMS is limited in experiments involving modulation of walking speed as subjects will have higher RMS when they walk faster [5,6]. In other words, it is necessary to consider the walking speed when RMS is used to assess gait abnormality.

It is generally known that some gait parameters exhibit common values or ratios in normal gait at an individual’s preferred walking speed (natural walking speed). For example, a walking cycle lasts around 1 s; stance and swing durations are consistently approximately 60% and 40% of a walking cycle in normal gait [18]. It is known that these parameters change with abnormal gait. For instance, Stolze et al. reported that a significantly increased walking cycle with a prolonged stance and double limb support duration was found in cerebellar ataxic gait [19]. We hypothesized (1) that the RMS values in three sensing axes also exhibit common proportions for healthy people if they walk at their own preferred speed and (2) that the RMS proportions in abnormal gait deviate from the common proportions. In this study, we propose the RMS ratio (RMSR described in the Analysis section) as a gait abnormality measure and verify its ability to discriminate abnormal gait.

## Methods

### Subjects

To examine the relationship between individual’s preferred walking speed and the RMSR, forty-seven healthy male subjects (age: 21–49 years; height: 172.5 ± 4.6 cm (mean ± S.D.); weight: 64.7 ± 8.6 kg) were recruited. None had a history of neurological, musculoskeletal or gait disorder, or any painful condition likely to affect their balance or mobility. Auvinet et al. reported that gait variables such as walking speed and characteristics of vertical acceleration started to change after 50 years of age for men [2]. Therefore, our condition of “healthy subjects” excludes people with age-related functional decline.

To verify the ability of RMSR to discriminate abnormal gait, twenty age-matched male post-stroke hemiplegic patients (age: 28–48 years; height: 170.8 ± 5.9 cm; weight: 65.9 ± 8.2 kg; Brunnstrom stage III: 3, IV: 9, V: 5 and VI: 3) participated as typical subjects with gait abnormality. In addition, an elderly male hemiplegic (age: 76 years; height: 163 cm; weight: 60 kg; Brunnstrom stage V) participated to confirm the changes of RMSR along with recovery in an acute period. Mazzà et al. described that a significant gender difference was found in the RMS at the pelvis [17]. To exclude an effect of gender, only male subjects and male patients were adopted in the experiment.

This study was approved by the ethics committees of Fujimoto General Hospital and Chiba University, and all the subjects gave written informed consent before examination.

### Data collection

A wireless sensor unit was used, which contained a tri-axial accelerometer (MMA7260Q, Freescale Semiconductor, USA), an infrared (IR) remote control receiver (NJL21H380A, New Japan Radio, Japan), a microcontroller and a Bluetooth module. This sensor unit was developed in our laboratory and its electronic design and characteristics permit a measuring range of ± 4 g, sensitivity of 0.002 g and a range of response frequency from 0 Hz to 30 Hz for the accelerometer. The accelerometer and IR receiver outputs were digitized at a sampling rate of 100 Hz by the microcontroller and sent to a computer via the Bluetooth module for further off-line analysis. The unit size and weight are 52 × 54 × 18 mm and 55 g including a battery, respectively.

To measure walking speed, we used a photoelectric sensor (PZ2-61, Keyence, Japan) connected to an IR LED circuit. When a subject passes between the sensor and its reflector, the sensor turns into the ON position, and then the ON signal emits IR. The sensor and the circuit were mounted on an upright pole. Two poles were placed at positions 2 m from the start and 2 m from the end of the walking path. The distance between the sensors was 10 m. The height of the sensors was adjusted to the height of the subject's shoulders. Walking speed between the photoelectric sensors was calculated by the distance and the IR LED emission timing detected by the IR remote control receiver built into the wireless sensor unit. The timing was also used to extract the acceleration signals associated with the walking speed.

### Procedure

The wireless sensor unit was attached to the lower back, around the L3-L4 vertebrae, using an elastic belt. This position was chosen owing to its proximity to the center of mass of the human body during standing. The sensing axes were oriented along the anatomical anteroposterior (AP), mediolateral (ML) and vertical (V) directions.

Forty-one of the healthy subjects and the patients were instructed to walk along a 14-m walking path at their own preferred speed. The healthy subjects wore their usual shoes, excluding low-heeled footwear such as sandals or slippers. If the hemiplegic patients usually used a cane and/or a short leg brace, they also used them in the measurement.

The measurement was carried out once per each subject after he walked along the walking path once to determine the optimal height of the photoelectric sensors for the individual subject. Only the elderly post-stroke hemiplegic patient carried out the measurement six times. Each measurement was performed after 13, 17, 21, 25, 31 and 40 days of the onset.

The other six healthy subjects were asked to walk at 0.83, 1.11, 1.39, 1.67 and 1.94 m/s on a treadmill for 2 min each.

### Analysis

The recorded acceleration signal consists of both a dynamic component reflecting changes in velocity during walking and a static acceleration caused by gravity. The sensing axes of the accelerometer may not be aligned with the axes of the horizontal-vertical coordinate system even if the sensor unit is attached to the human body carefully. This discordance of axes introduces small calculation errors. To remove the static component and transform the measured dynamic accelerations into the horizontal-vertical coordinate system, the algorithm proposed by Moe-Nilssen [20] was applied to the recorded acceleration signal. This algorithm is based on a simple trigonometric computation and transforms by using the acceleration signal reflecting the tilt angle of the sensor under static conditions.

After corrections of the signal, RMS and RMSR were calculated using the following equations:

(1) $$\mathrm{RM}S_{T}=\sqrt{\mathrm{RM}{S_{\mathrm{AP}}}^{2}+\mathrm{RM}{S_{\mathrm{ML}}}^{2}+\mathrm{RM}{S_{v}}^{2}}$$

(2) $$\mathrm{RMS}R_{x}=\mathrm{RM}S_{x}/\mathrm{RM}S_{T}$$

where *x* represents the direction of acceleration. The RMS is a statistical measure of the magnitude of acceleration in each direction. In this study, the RMS coincides with the standard deviation since the acceleration signals were transformed to give a mean equal to zero. The RMSR represents the ratio between RMS in each direction and the RMS vector magnitude (*RMS*_*T*_). In other words, the RMSR is the RMS normalized by the *RMS*_*T*_.

Some researchers have reported that the RMS demonstrated a non-linear relationship to walking speed [3,4,15]. Therefore, Spearman’s rank correlation coefficient *r*_*s*_ was used to evaluate the relationship among gait parameters and subject characteristics. This coefficient is a non-parametric measure of statistical dependence between two variables. For RMS and RMSR, Mann-Whitney’s *U* Test was used to test the difference between the healthy subjects and the hemiplegic subjects.

## Results

**Figure 1:**
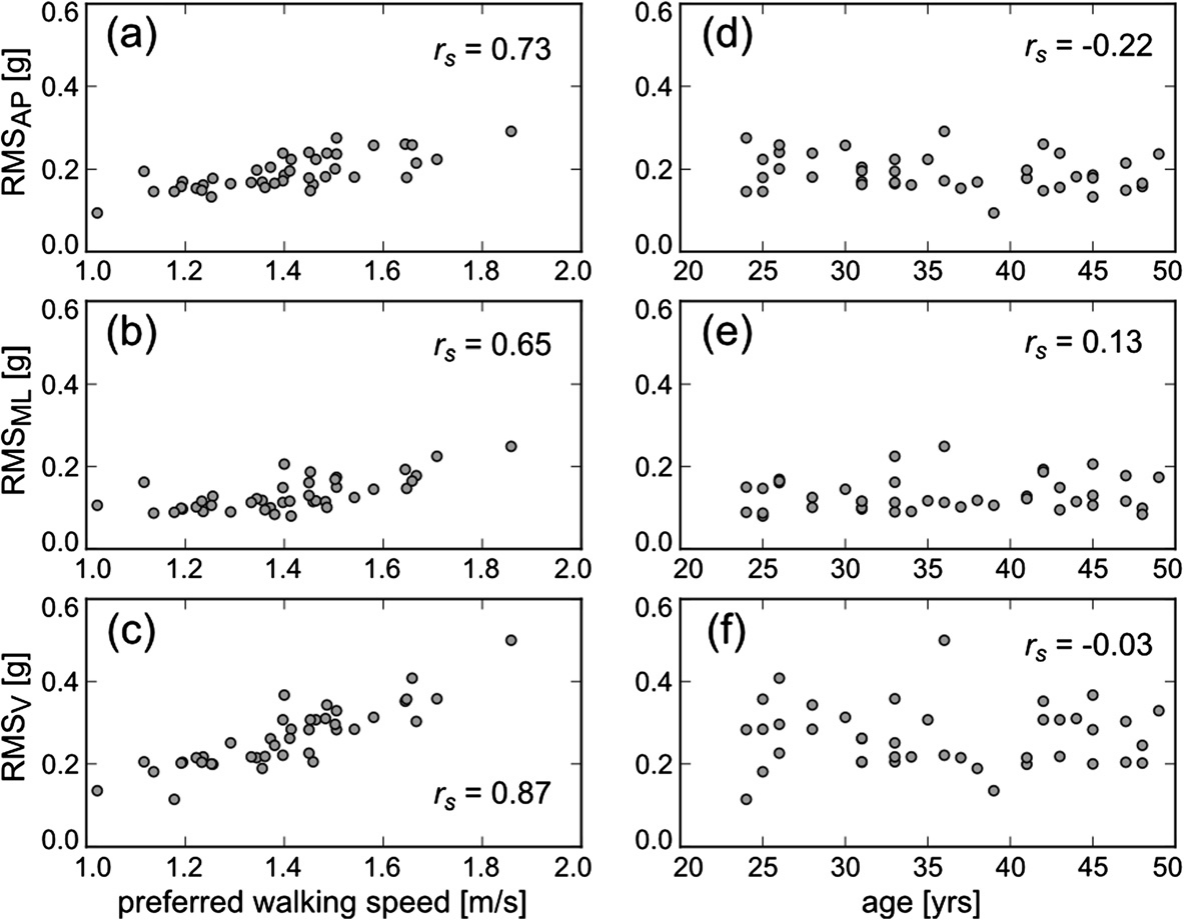
**The effect of preferred walking speed on RMS in AP (a), ML (b) and V directions (c), and the effect of age on RMS in AP (d), ML (e) and V directions (f).** Each circle shows the RMS of each healthy subject at his preferred walking speed. *r*_*s*_ represents Spearman’s rank correlation coefficients.

**Figure 2:**
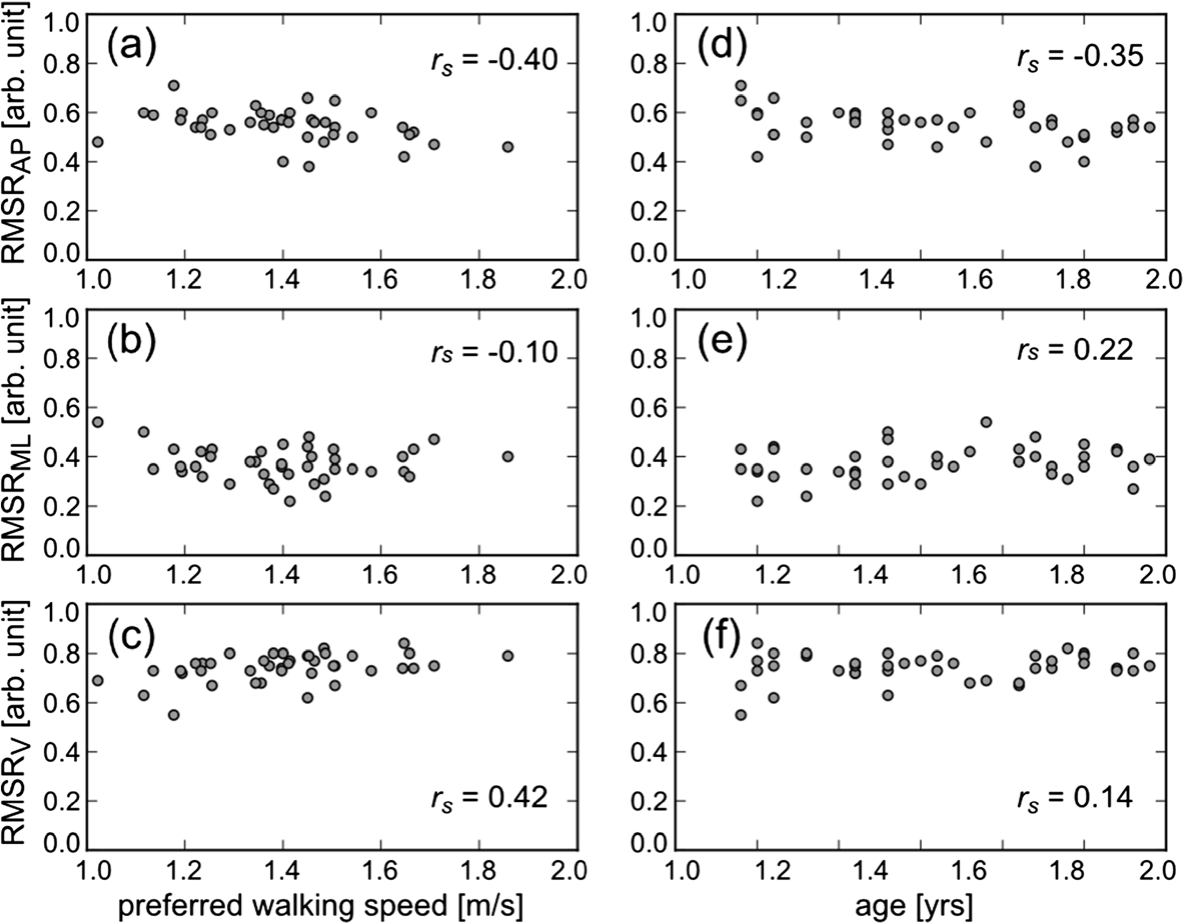
**The effect of preferred walking speed on RMS ratio (RMSR) in AP (a), ML (b) and V directions (c), and the effect of age on RMSR in AP (d), ML (e) and V directions (f).** Each circle shows the RMSR of each healthy subject at his preferred walking speed. *r*_*s*_ represents Spearman’s rank correlation coefficients.

Figure 1 shows the relationship between preferred walking speed and the acceleration RMS in the healthy subjects. The range of preferred walking speed was 1.02 - 1.86 m/s. This result means that the fastest subject selected a preferred walking speed nearly twice that of the slowest subject. The acceleration RMS in all directions increased as the preferred walking speed became faster. The Spearman's rank correlation coefficients between preferred walking speed and the RMS values in AP, ML and V directions were 0.73 (*p* < 0.01), 0.65 (*p* < 0.01) and 0.87 (*p* < 0.01), respectively. In contrast, there were no strong relationships between age and the RMS values in all directions (AP: *r*_*s*_ = -0.22, *p* = 0.16; ML: *r*_*s*_ = 0.13, *p* = 0.42; V: *r*_*s*_ = -0.03, *p* = 0.86). On the other hand, although the RMSR in AP and V directions were still influenced by preferred walking speed (AP: *r*_*s*_ = -0.40, *p* < 0.01; V: *r*_*s*_ = 0.42, *p* < 0.01), the RMSR in the ML direction did not correlate with preferred walking speed (*r*_*s*_ = -0.10, *p* = 0.54), as shown in Figure 2. In addition, there was no strong relationship between age and RMSR in the ML direction (*r*_*s*_ = 0.22, *p* = 0.17). The mean ± SD of RMSR in the ML direction was 0.37 ± 0.07 in the healthy subjects.

**Figure 3:**
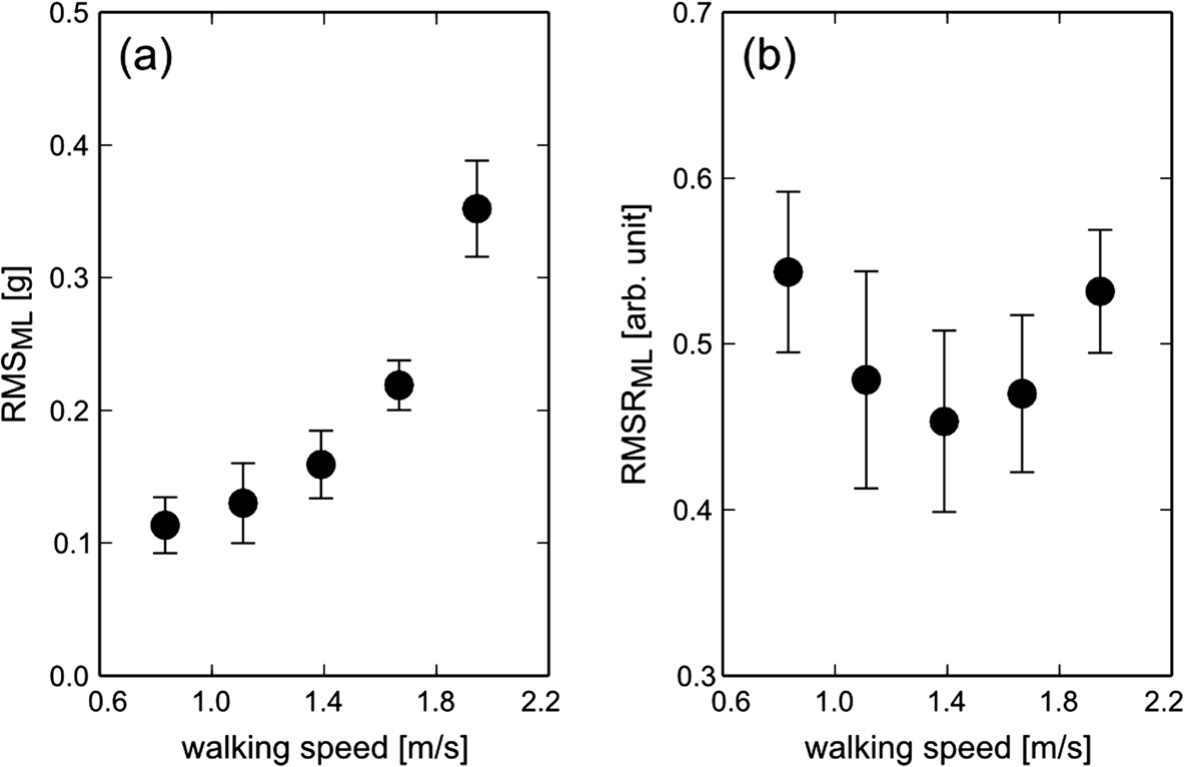
The effect of controlled walking speed on RMS (a) and RMSR (b) in the ML direction for the healthy subjects.

When the six healthy subjects were instructed to walk at the five controlled speeds from 0.83 to 1.94 m/s, the RMS in the ML direction showed an exponential relationship with walking speed (Figure 3). This is consistent with previous studies [3-6,11,15]. On the other hand, the RMSR in the ML direction showed a U-shaped relationship to walking speed. The minimum value of the RMSR was 0.45 at 1.39 m/s. After the experiment, all subjects answered that the preferred speed was 1.39 m/s among the five controlled speeds. These results indicate that the RMSR is minimal at the preferred walking speed, and increases with faster or slower walking.

**Figure 4:**
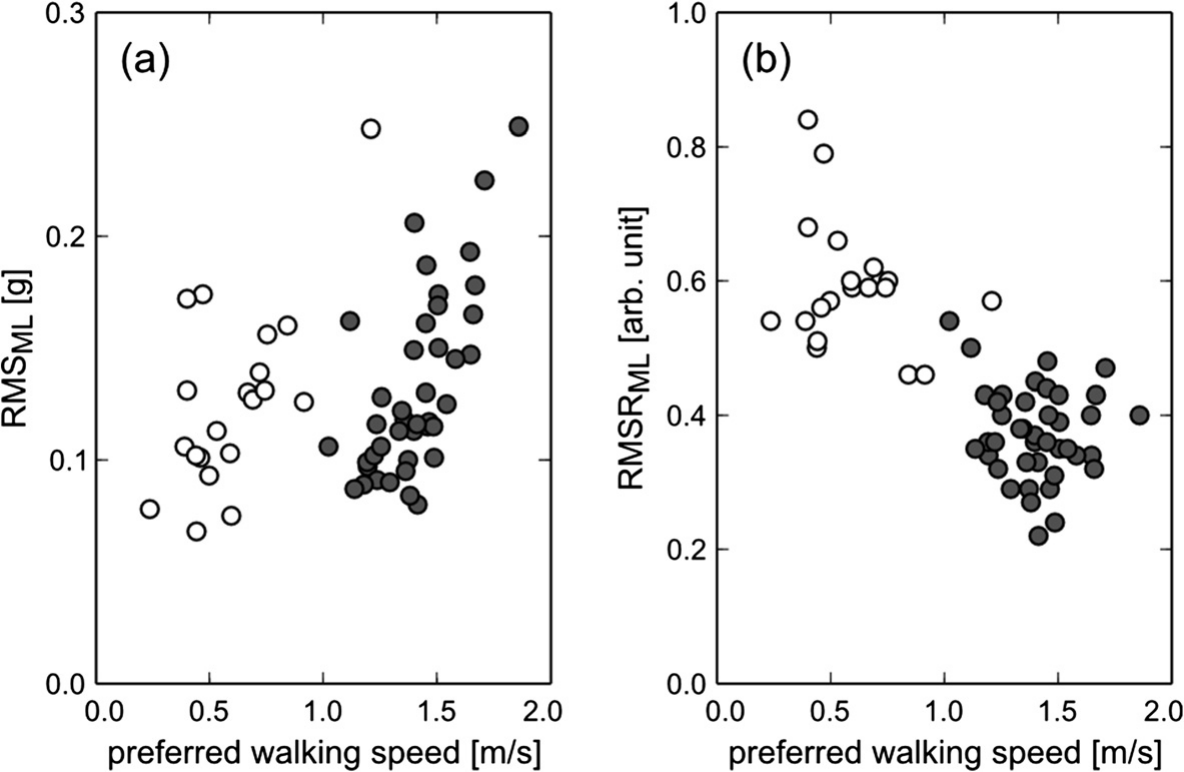
**The effect of preferred walking speed on RMS (a) and RMSR (b) in the ML direction for the healthy subjects and hemiplegic patients.** Each shaded circle represents the value of each normal subject, and each open circle represents the value of each the hemiplegic patient. The values in the healthy subjects are the same as in Figures 1 and 2.

Figure 4 shows RMS and RMSR values in the ML direction for the healthy subjects and the age-matched hemiplegic patients. The values in the healthy subjects were the same as in Figures 1 and 2. In the hemiplegic patients, the range of preferred walking speed was 0.24 - 1.21 m/s. The hemiplegic patients selected a significantly slower walking speed than the healthy subjects (p < 0.01); however, there was no significant decrease in the RMS of the hemiplegic patients compared with that in healthy subjects (*p* = 0.23). These results indicate that RMS was influenced by gait abnormality. On the other hand, the RMSR in the ML direction for the hemiplegic patients was 0.59 ± 0.09, which was significantly higher than that in the healthy subjects (*p* < 0.01).

**Figure 5:**
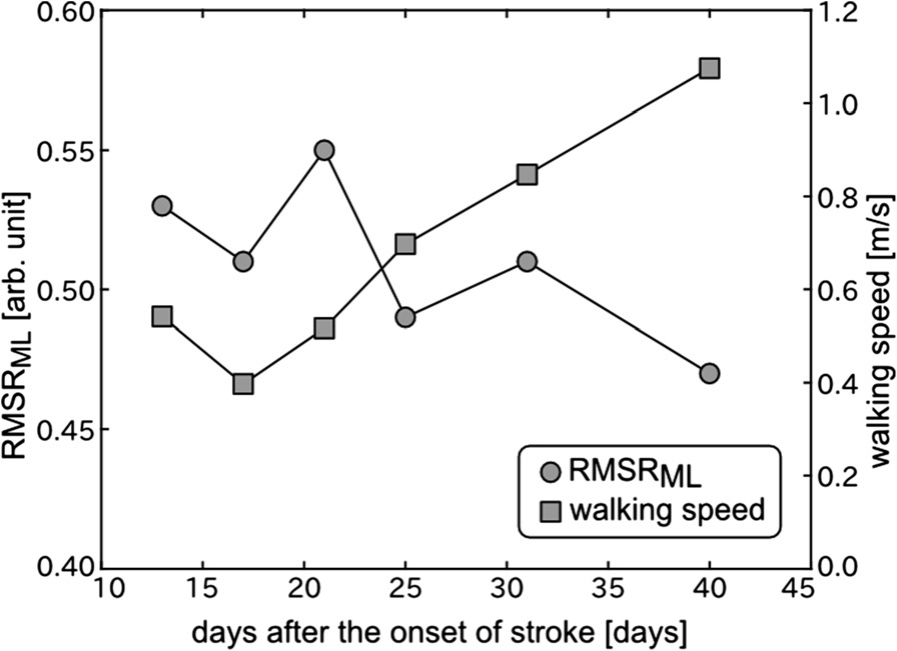
**Changes in walking speed and the RMS ratio (RMSR) in the acute period of an elderly hemiplegic patient.** Circles represent the RMSR in the ML direction, and squares represent walking speed.

Figure 5 shows the changes in walking speed and the RMSR in the ML direction in an acute period of the elderly hemiplegic patient. The walking speed increased along with the recovery from motor disorder by physical therapy. The RMSR decreased while slightly fluctuating and reached its minimum value on the last measurement day.

## Discussion

In this study, a new measure, RMSR, was proposed to evaluate gait abnormality. Our major finding is that acceleration RMSR in the ML direction represented the similar value in the healthy subjects when they walked at their own preferred speed, even if their preferred walking speed were different, as shown in Figure 2(b). This finding suggests that the RMSR in the ML direction exhibits a common value in normal gait at an individual’s preferred walking speed like walking cycle, stance and swing durations. In addition, although RMSR in AP and V directions were influenced by preferred walking speed, RMSR in the sagittal plane also adopts a common value, theoretically. This is because *RMSR*_*ML*_^2^ + *RMSR*_*s*_^2^ = 1, where $\mathrm{RMS}R_{S}=\sqrt{\mathrm{RMS}{R_{\mathrm{AP}}}^{2}+\mathrm{RMS}{R_{V}}^{2}}$ represents RMSR in the sagittal plane.

During walking, the gravitational potential energy of the center of body mass is exchanged for forward kinetic energy [21]. Thus, forward motion is mainly caused by AP and V movements in the sagittal plane. ML movement plays only a small role in external work [21]. The acceleration RMS in the ML direction increased in a slightly balance-impaired subject compared with that in normal subjects, when they walked at the same speed [4]. Although the measurement site was slightly different from that in our study, Menz et al. reported that walking on an irregular surface resulted in significant increases in acceleration RMS at the pelvis when subjects walked on the irregular surface while maintaining their preferred speed [5]. When the RMSR was calculated from the RMS values shown in their study, the RMSR in the ML direction increased under the irregular surface conditions. Therefore, we speculate that the RMSR in the ML direction are primarily associated with walking balance. If the subjects are permitted to choose their own speed, they will select a speed that minimizes the energy cost per distance [22,23]. In addition, walking faster or slower requires more energy per step [22,23]. These factors suggest that subjects optimize their body movement when they walk at their own preferred speed. These findings are similar to the relationship between RMSR and walking speed, as shown in Figure 3. The RMSR was minimized at the preferred walking speed, and increased at a faster or slower walking speed. As a result of such body movement optimization, it was considered that the RMSR in the sagittal plane and the ML direction converged to the common values in normal gait.

To confirm that the RMSR in the ML direction can be used as a gait abnormality measure, the age-matched post-stroke hemiplegic patients were evaluated as typical subjects with gait abnormality. Compared with the healthy subjects, the RMSR in the ML direction significantly increased in the hemiplegic patients, although there was no significant difference of the RMS value in ML direction between the healthy subjects and the hemiplegic subjects. This result suggests that it is difficult to assess gait abnormality by the directly use of the RMS value in ML direction. It is known that almost all hemiplegic patients walk slower than healthy people [16]. In fact, the hemiplegic patients selected a slower walking speed than the healthy subjects in this study. Acceleration RMS was closely associated with preferred walking speed in the healthy subjects, as shown in Figure 1(a)-(c). If the same relationship holds true for hemiplegic patients, the RMS value should be small. Our results suggested that the RMS value is related to not only walking speed but also gait abnormality. Moreover, it was actually confirmed, as shown in Figure 5, that the RMSR in the ML direction decreased along with recovery in the acute period, although this was a single-case experiment. Changes in the RMSR and walking speed showed different patterns in terms of the time courses. As described above, the RMSR in the ML direction exhibited a common value when the normal subjects walked at their own preferred speed. Therefore, the increased RMSR in the ML direction represents gait abnormality. Hemiplegic stroke patients frequently present gait abnormalities because of decreased muscle strength, range of movement, abnormal muscle tone, motor coordination, sensory organization, cognition, and multisensory integration [24]. The RMSR can not specify the cause of abnormal gait. However, the results indicate that it is possible to evaluate comprehensive gait abnormality and to provide objective evidence for treatment outcome during rehabilitation by the RMSR in the ML direction.

Moe-Nilssen compared RMS values in the ML direction between normal subjects and a subject with slight balance impairment at the same walking speed [4]. However, it might be difficult to unite the walking speeds in clinical practice. For stroke patients, Mizuike et al. normalized RMS by the square of the walking speed and multiplied it by the average step length to exclude the effect of walking speed [16]. Although there was no description of the relationship between the normalized RMS and walking speed, they suggested that the normalized RMS might reflect the degree of functional recovery after stroke better than raw RMS. By taking into account walking speed, the RMS value can be used for indicator of the smoothness or dynamics of the walking pattern [16]. If walking speed is included as a computation variable and is measured accurately, it requires other equipment such as a photoelectric sensor, as described in the Data collection section. The advantage of the RMSR is that this value can be calculated using only the tri-axial acceleration and provide information of gait abnormality considering the difference of individual walking speed. The RMSR takes its minimum value at the individual preferred walking speed for each subject, and increases at abnormal gait. By only involving the instruction to walk at a preferred walking speed, it is simple, but can assess abnormality of gait. The convenience of RMSR may make it useful as a screening marker of gait abnormality and a record of recovery from impairment for practical use.

## Conclusions

In this study, we demonstrated that the acceleration RMSR in the ML direction exhibits a common value in healthy subjects under the condition that they walk at their own preferred speed. We also showed that this RMSR in the ML direction is increased in hemiplegic patients with gait abnormality under the same condition. In addition, although the experiment during acute period was done for only one hemiplegic patient, the process of recovery from impairment could be quantified with the RMSR in the ML direction. The RMSR can be computed only by measuring walking at a subject’s own preferred speed using a tiny accelerometer, and may potentially be a quantitative measure of gait abnormality.

## Competing interests

The authors declare that they have no competing interests.

## Authors’ contributions

MS performed the design of the study, measurements of healthy subjects, data analysis, and drafted the manuscript. TT and MY were involved in interpretation of results and critical revision of the manuscript for important intellectual content. YS participated in the measurements of healthy subjects, data analysis, and drafted the manuscript. YK was involved in interpretation of results and drafting the manuscript. HM participated in the measurements of healthy subjects and drafted the manuscript. YK and YH performed the measurements of hemiplegic patients and assisted with drafting the manuscript. TF coordinated the study and assisted with drafting the manuscript. All authors read and approved the final manuscript.
